# A key role of miR-132-5p in the prefrontal cortex for persistent prophylactic actions of (*R*)-ketamine in mice

**DOI:** 10.1038/s41398-022-02192-6

**Published:** 2022-09-28

**Authors:** Li Ma, Long Wang, Lijia Chang, Jiajing Shan, Youge Qu, Xingming Wang, Xiayun Wan, Yuko Fujita, Kenji Hashimoto

**Affiliations:** 1 Division of Clinical Neuroscience, Chiba University Center for Forensic Mental Health, Chiba, Japan; 2 Department of Anesthesiology, Renmin Hospital of Wuhan University, Wuhan, Hubei Province China

**Keywords:** Depression, Pharmacodynamics

## Abstract

(*R,S*)-ketamine is known to elicit persistent prophylactic effects in rodent models of depression. However, the precise molecular mechanisms underlying its action remain elusive. Using RNA-sequencing analysis, we searched for novel molecular target(s) that contribute to the prophylactic effects of (*R*)-ketamine, a more potent enantiomer of (*R,S*)-ketamine in chronic restraint stress (CRS) model. Pretreatment with (*R*)-ketamine (10 mg/kg, 1 day before CRS) significantly ameliorated body weight loss, increased immobility time of forced swimming test, and decreased sucrose preference of sucrose preference test in CRS-exposed mice. RNA-sequencing analysis of prefrontal cortex (PFC) revealed that several miRNAs such as miR-132-5p might contribute to sustained prophylactic effects of (*R*)-ketamine. Methyl CpG binding protein 2 (MeCP2) is known to regulate brain-derived neurotrophic factor (BDNF) expression. Quantitative RT-PCR confirmed that (*R*)-ketamine significantly attenuated altered expression of *miR-132-5p* and its regulated genes (*Bdnf, Mecp2, Tgfb1, Tgfbr2*) in the PFC of CRS-exposed mice. Furthermore, (*R*)-ketamine significantly attenuated altered expression of BDNF, MeCP2, TGF-β1 (transforming growth factor β1), and synaptic proteins (PSD-95, and GluA1) in the PFC of CRS-exposed mice. Administration of agomiR-132-5p decreased the expression of *Bdnf* and *Tgfb1* in the PFC, resulting in depression-like behaviors. In contrast, administration of antagomiR-132-5p blocked the increased expression of *miR-132-5p* and decreased expression of *Bdnf* in the PFC of CRS-exposed mice, resulting in antidepressant-like effects. In conclusion, our data show a novel role of miR-132-5p in the PFC underlying depression-like phenotypes in CRS model and the sustained prophylactic effects of (*R*)-ketamine.

## Introduction

The *N*-methyl-D-aspartate receptor (NMDAR) antagonist (*R,S*)-ketamine is a promising drug in the treatment of severe depression. In 2000, Berman et al. [1] reported the rapid and sustained antidepressant actions of (*R,S*)-ketamine in medication-free patients with major depressive disorder (MDD). Subsequent numerous studies have replicated the robust antidepressant effects of (*R,S*)-ketamine in treatment-resistant patients with MDD, bipolar disorder (BD), or post-traumatic stress disorder (PTSD) [2–8]. In addition, (*R,S*)-ketamine rapidly reduces suicidal thoughts in depressed patients with suicidal ideation [9, 10]. Although (*R,S*)-ketamine can elicit the potent antidepressant and anti-suicidal actions in patients with severe depression, the precise molecular mechanisms underlying its actions remain largely unclear [11–19].

In addition to robust antidepressant actions, (*R,S*)-ketamine causes persistent prophylactic effects against chronic social defeat stress (CSDS) model, learned helplessness (LH) model, chronic corticosterone-treated model, and lipopolysaccharide (LPS)-induced inflammation model [20–22]. Notably, Ma et al. [23] reported prophylactic effects of (*R,S*)-ketamine on postpartum depression in Chinese women undergoing cesarean section. Collectively, it is likely that (*R,S*)-ketamine could show prophylactic effects against stress-related psychiatric disorders such as MDD. However, the precise molecular and cellular mechanisms underlying prophylactic actions of (*R,S*)-ketamine remain unclear.

(*R,S*)-ketamine is a racemic mixture that contains equal amounts of (*R*)-ketamine (or arketamine) and (*S*)-ketamine (or esketamine), with (*S*)-ketamine having a higher affinity at NMDAR. In 2019, (*S*)-ketamine nasal spray for treatment-resistant MDD patients was approved in the United State and Europe. In contrast, accumulating evidence shows that (*R*)-ketamine displays greater potency and longer-lasting antidepressant-like effects than (*S*)-ketamine in rodent models of depression [24–34], suggesting that NMDAR does not play a major role in the robust antidepressant-like effects of (*R,S*)-ketamine [11–15, 35, 36]. Importantly, side effects of (*R*)-ketamine are lower than those of (*R,S*)-ketamine and (*S*)-ketamine [25, 31, 37–39]. A recent study shows that abuse liability of (*R,S*)-ketamine in humans is primarily due to pharmacological effects of (*S*)-ketamine, but not (*R*)-ketamine [40]. A pilot study showed that (*R*)-ketamine caused rapid-onset and sustained antidepressant actions in treatment-resistant patients with MDD, and that side effects such as dissociation were very low [41]. Collectively, (*R*)-ketamine would be a novel antidepressant without side effects of (*R,S*)-ketamine [11, 14, 17, 42, 43]. Recently, we reported that nuclear factor of activated T cells 4 in the prefrontal cortex (PFC) plays a role in persistent prophylactic effects of (*R*)-ketamine in LPS-induced inflammation model of depression [44]. However, there are no articles reporting the prophylactic effects of (*R*)-ketamine in rodents exposed to chronic stress. In addition, the precise molecular mechanisms underlying the prophylactic effects of (*R*)-ketamine remain unclear.

Small non-coding RNAs known as microRNAs (or miRNAs) can regulate gene expression in the body, and miRNAs are critical regulators of brain development, brain function, and diseases [45–48]. It is known that miRNAs are detected in the brain and body fluids of human. Interestingly, blood levels of miRNAs in patients with MDD are altered compared to healthy control subjects [49, 50]. Thus, it is likely that circulatory miRNAs may be potential diagnostic and therapeutic biomarkers for depression.

The aim of this study was to identify the novel molecular mechanisms underlying the sustained prophylactic effects of (*R*)-ketamine in chronic restrain stress (CRS) model which is widely used to recapitulate depression-like behaviors including anhedonia in rodents [51]. First, we performed RNA-sequencing analysis of PFC of CRS-exposed mice treated with either (*R*)-ketamine or saline, as PFC contributes to the antidepressant-like actions of ketamine and its enantiomers [52, 53]. In this study, we focused non-coding RNAs known as microRNAs (miRNAs) in the PFC since miRNAs play a role in the antidepressant-like effects of (*R,S*)-ketamine in rodents exposed to chronic stress [54, 55]. Furthermore, we examined the role of miRNAs in the sustained prophylactic effects of (*R*)-ketamine in CRS-exposed mice.

## Materials and method

### Animals

Adult male C57BL/6 mice (aged 8 weeks old, weighed 20–25 g) were purchased from Japan SLC Inc. (Hamamatsu, Japan). Mice were housed under controlled conditions for temperature and humidity with a 12-hour light/dark cycle (lights on from 7:00 to 19:00) and were allowed free access to food (CE-2; CLEA Japan, Inc., Tokyo, Japan) and water. All experiments using mice were carried out in strict accordance with the recommendations in the Guide for the Care and Use of Laboratory Animals of the National Institutes of Health, USA, and were approved by the Chiba University Institutional Animal Care and Use Committee (Permission number: 1–448, 3–53, 4–221, and 4–374). Animals were deeply anaesthetized with inhaled isoflurane (2–5 vol%) and rapidly sacrificed by cervical dislocation. All efforts were made to minimize animal suffering.

### Compounds and treatment

(*R*)-ketamine hydrochloride was prepared by recrystallization of (*R,S*)-ketamine (Ketalar^®^, ketamine hydrochloride, Daiichi Sankyo Pharmaceutical Ltd., Tokyo, Japan) and D-(-)-tartaric acid, as reported previously [24]. (*R*)-norketamine hydrochloride was synthesized as reported previously [26]. (2 *R*,6 *R*)-hydroxynorketamine (HNK) hydrochloride was purchased from Tocris Bioscience (Tokyo, Japan). The dose (10 mg/kg as hydrochloride salt) of (*R*)-ketamine, (*R*)-norketamine, and (2 *R*,6 *R*)-HNK were selected as reported previously [25, 28–30, 44]. The mmu-miR-132-5p miRNA agomir/antagomir (catalog number: AM3000, AcceGen Biotech, Fairfield, USA) was dissolved in diethypyrocarbonate (DEPC) water. The dose (200 nmol) and the route [intracerebroventricular (i.c.v.) injection] of administration of mmu-miR-132-5p miRNA agomir/antagomir were followed by the manufacture’s protocol. Under isoflurane anesthesia, mmu-miR-132-5p miRNA agomir/antagomir (200 nmol/day for 3 consecutive days) was administered i.c.v. to mouse brain.

### Chronic restraint stress (CRS) model and behavioral tests

**Fig. 1:**
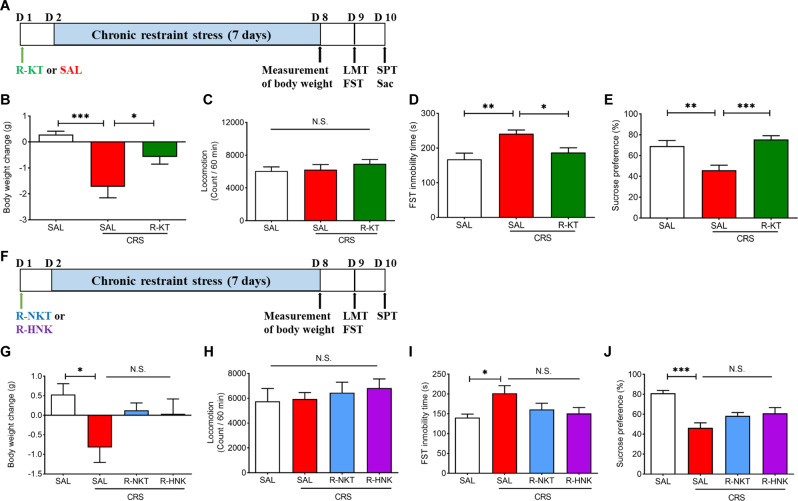
Prophylactic effects of (*R*)-ketamine, not its metabolites, on CRS model of depression. **A** Treatment schedule. (*R*)-ketamine (10 mg/kg) or saline (10 ml/kg) was i.p. injected to adult mice 1 day before chronic restraint stress (CRS) (day 1). Subsequently, mice were subjected to CRS for 7 days (day 2–day 8). Locomotion test (LMT), forced swimming test (FST) and sucrose preference test (SPT) were performed on day 9 and day 10, respectively. The prefrontal cortex (PFC) was collected after the SPT (day 10). **B** Body weight change (one way ANOVA: F_2,27_ = 11.02, *P* = 0.0003). **C** Locomotion test (one way ANOVA: F_2,27_ = 0.648, *P* = 0.531). **D** FST (one way ANOVA: F_2,27_ = 7.793, *P* = 0.002). **E** SPT (one way ANOVA: F_2,27_ = 10.75, *P* = 0.0004). The data represent mean ± SEM (*n* = 9–11). *P < 0.05, **P < 0.01, ***P < 0.0001. N.S., not significant. **F** Treatment schedule. (*R*)-norketamine (R-NKT; 10 mg/kg), (2 *R*,6 *R*)-hydroxynorketamine (R-HNK; 10 mg/kg) or saline (10 ml/kg) was i.p. injected to mice 1 day before CRS (day 1). Subsequently, mice were subjected to CRS for 7 days (day 2 -day 8). LMT, FST, and SPT were performed on day 9 and day 10, respectively. **G** Body weight change (one way ANOVA: F_3,28_ = 3.221, *P* = 0.038). **H** Locomotion test (one way ANOVA: F_3,28_ = 0.3635, *P* = 0.780). **I** FST (one way ANOVA: F_3,28_ = 3.171, *P* = 0.040). **J** SPT (one way ANOVA: F_3,28_ = 10.59, *P* < 0.0001). The data represent mean ± SEM (*n* = 8). *P < 0.05, ***P < 0.001. N.S., not significant.

Mice were bound to restraint well-ventilated 50-mL plastic tubes without food and water from 17:00 PM to 19:00 PM for 7 consecutive days, they could not move freely or turn around but without oversqueezed; this procedure induces chronic stress without pain or injury. After being restrained, mice were released back to their home cage immediately. Mice without restrained were remained in their usual environment and were undisturbed for 7 days. Saline (10 ml/kg) or (*R*)-ketamine (10 mg/kg) was given intraperitoneally (i.p.) to mice 1 day before being subjected to CRS (Fig. 1A).

The locomotion (LMT), forced swimming test (FST) and sucrose preference test (SPT) were performed as described previously [30–32, 44, 56]. An automated animal movement analysis system (SCANET MV-40; MELQUEST Co., Ltd, Toyama, Japan) was used to measure the locomotor activity of each mouse. The cumulative ambulatory activity counts were recorded continuously over a period of 60 minutes after the mice were placed in the experimental cages (56 cm (length) × 56 cm (width) × 33 cm (height)). The cages were cleaned between the testing sessions. The FST was performed using an automated forced-swim apparatus (SCANET MV-40; MELQUEST Co., Ltd, Toyama, Japan). The mice were individually placed into a cylinder (23 cm (diameter) × 31 cm (height)) with a water depth of 15 cm (water temperature, 23 ± 1 °C). The immobility time was recorded and calculated by the analytical software of the apparatus throughout a 6-minute observation time. Mice were exposed to water and 1% sucrose solution for 48 h, followed by 4 h of water and food deprivation and a 1-hour exposure to two identical bottles, one is water, and another is 1% sucrose solution. The bottles containing water and sucrose were weighed before and at the end of this period. The sucrose preference was calculated as a percentage of sucrose solution consumption to the total liquid consumption.

### RNA-sequencing analysis and Quantitative real-time PCR

PFC was collected 2 days after CRS. Total RNA isolated from PFC obtained from saline (10 ml/kg) + CRS group (*n* = 5) and (*R*)-ketamine (10 mg/kg) + CRS group (*n* = 5) was used for RNA-sequencing analysis. RNA-sequencing analysis was performed at the Novogene Company (Beijing, China).

A quantitative RT-PCR system (Step One Plus, Thermo Fisher Scientific, Yokohama, Japan) was used. All specific mRNA transcripts were quantitatively analyzed by TaqManGene Expression assays (Thermo Fisher Scientific, Yokohama, Japan). The gene expression levels of *Bdnf* (Mm04230607_s1), *Mecp2* (Mm01193537_g1), *Tgfb1* (Mm01178820_m1), *Tgfbr1* (Mm00436964_m1) were measured. Total RNA was extracted using an RNase-Free DNase Set and a RNeasy Mini Kit (Qiagen, Hilden, Germany). The purity of total RNA was assessed by Bio photometer plus (Eppendorf, Hamburg, Germany). The cDNA libraries were obtained by reverse transcription-PCR using a High-Capacity cDNA Reverse Transcription Kit (catalog number: #4368813 Thermo Fisher Scientific, Yokohama, Japan). All specimens were detected twice, and arithmetic means were used for quantification. The data of arithmetic mean were normalized to Vic-labeled Actb mRNA (catalog number: #4352341E: pre-developed TaqMan Assay Reagents, Thermo Fisher Scientific, Yokohama, Japan).

### Measurement of miRNAs in the PFC

The total RNA was extracted by miRNeasy mini kit (Qigen, Hilden, Germany) following the manufacturer’s instructions. RNA quality and quantity were determined by Bio photometer plus (Eppendorf, Hamburg, Germany). cDNA synthesis from miRNAs was obtained using the Taqman Advanced miRNA cDNA Synthesis Kit (Cat. No. A28007). RNA concentration should be ≤ 5 ng/µL. First, strand cDNA synthesis was performed the poly(A) tailing. Second strand cDNA synthesis was performed the adaptor ligation. Third adaptor-cDNA was performed reverse transcription (RT) reaction. At last RT-reaction cDNA was performed the miR-Amp reaction. The miR-132-5p (mmu481539_mir: catalog number: #A25576, Thermo Fisher Scientific, Yokohama, Japan) and snoRNA-202 (catalog number: #001232, Thermo Fisher Scientific) cDNA was used with TaqMan fast advanced master mix (catalog number: #4444557, Thermo Fisher Scientific) for real-time PCR. The expression of miRNA was measured with StepOne Plus™ (Thermo Fisher Scientific) according to the manufacturer’s instructions. Each cDNA sample was tested in duplicate (as in comparable studies and dCT was determined by normalization of the Ct value to the Ct value of the reference small nucleolar RNA snoRNA-202 for miRNA. Data were transformed into relative values by calculating: 2 − ΔΔCT (ddCT).

### Western blotting

Western blot analysis was performed as previously reported [44, 57]. Tissue samples from the PFC were homogenized in ice-cold Laemmli lysis buffer and centrifuged at 3000 × *g* for 10 min at 4 °C, to collect the supernatants. Proteins were quantified using a bicinchoninic acid protein assay kit (Bio-Rad, Hercules, CA). The samples were then mixed with an equal volume of loading buffer (125 mM Tris/HCl, pH 6.8, 20% glycerol, 0.1% bromophenol blue, 10% β-mercaptoethanol, and 4% sodium dodecyl sulfate) and boiled for 5 minutes at 95 °C. Proteins were separated using 10% sodium dodecyl sulfate polyacrylamide gel electrophoresis (SDS–PAGE) (Mini-PROTEAN^®^ TGX™ Precast Gel; Bio-Rad) and then transferred onto polyvinylidene difluoride membranes using a Trans Blot Mini Cell apparatus (Bio-Rad). The membranes were blocked with 5% skim milk in TBS containing 0.1% Tween 20 (TBST) for 1 h at room temperature, followed by incubation with primary antibodies against PSD-95 (1:1000, catalog number: 51-6900, Invitrogen, Camarillo, CA, USA), GluA1 (1 μg/mL, catalog number: ab31232, Abcam, Cambridge, MA, USA), BDNF (1:1,000, catalog number: A1307, ABclonal, Inc., Tokyo, Japan), MeCP2 (1:1,000, catalog number: M9317, Sigma-Aldrich Co., Ltd, St Louis, MO, USA), TGF-β1 (1:1,000, catalog number: SAB4502954, Sigma-Aldrich Co., Ltd, St Louis, MO, USA), and β-actin (1:10,000, Sigma-Aldrich Co., Ltd, St Louis, MO, USA) overnight at 4 °C. After three washes with TBST, the membranes were incubated with a horseradish peroxidase-conjugated anti-rabbit or anti-mouse antibody (1:5000) for 1 hour at room temperature. After three washes in TBST, the bands were visualized using enhanced chemiluminescence plus the Western Blotting Detection system (GE Healthcare Bioscience) and captured by a ChemiDoc™ Touch Imaging System (170-01401; Bio-Rad Laboratories, Hercules, CA). The images were subjected to grey-scale analysis using the Image LabTM 3.0 software (Bio-Rad Laboratories).

### Statistical analysis

The data were shown as mean ± standard error of the mean (SEM). Analysis was performed using PASW Statistics 20 (formerly SPSS Statistics; SPSS). The data were analyzed using the one-way analysis of variance (ANOVA), followed by *post-hoc* Tukey test. The data of body weight were analyzed by repeated measures two-way ANOVA followed by the Holm-Sidak’s post hoc test. The *P*-values of less than 0.05 were considered statistically significant.

## Results

### Prophylactic effects of (*R*)-ketamine and its metabolites on depression-like phenotypes in CRS model

To identify the prophylactic effects of (*R*)-ketamine in CRS-induced depression, saline or (*R)*-ketamine (10 mg/kg) was administered to mice 1 day before the start of CRS (7 days) (Fig. 1A). The body weight of mice was significantly decreased after CRS (Fig. 1B). Pretreatment with (*R*)-ketamine significantly attenuated CRS-induced body weight loss (Fig. 1B). There were no significant changes in the locomotor activity among the three groups (Fig. 1C). Pretreatment with (*R*)-ketamine significantly ameliorated CRS-induced increase in the immobility time of FST, and CRS-induced decreased sucrose preference in the SPT (Fig. 1D, E). In contrast, pretreatment with (*R*)-norketamine (10 mg/kg) or (2 *R*,6 *R*)-HNK (10 mg/kg), two major metabolites of (*R*)-ketamine, did not show prophylactic effects for the body weight loss and depression-like behaviors in CRS-exposed mice (Fig. 1F–J). Although it looks that two metabolites reversed slightly CRS-induced body weight loss in mice, statistical analysis did not react to significant difference (Fig. 1G).

### Prophylactic effects of (*R*)-ketamine on differentially expressed miRNAs in CRS-exposed mice

**Fig. 2:**
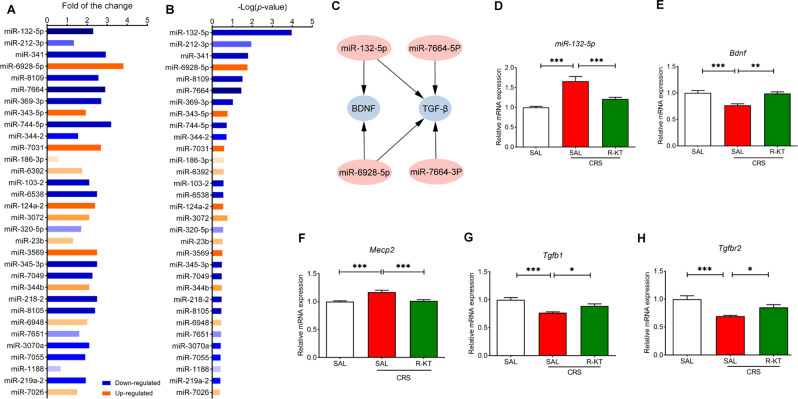
List of miRNAs and prophylactic effects of (*R*)-ketamine on miR-132-5p and related genes in the PFC from CRS mice. **A**, **B** RNA-sequence analysis of the prefrontal cortex (PFC) of CRS-exposed mice treated with (*R*)-ketamine (10 mg/kg) (*n* = 5) or saline (*n* = 5). The differentially expressed miRNAs were shown in the (**A**: fold of the change) and (**B**: *P* value). **C** Bioinformatics prediction by TargetScanMouse showed that miR-132-5p can regulate BDNF and TGF-β1. **D** Expression of *miR-132-5p* in the PFC (one way ANOVA: F_2,28_ = 19.27, *P* < 0.0001). **E** *Bdnf* mRNA in the PFC (one way ANOVA: F_2,28_ = 13.34, *P* < 0.0001). **F** *Mecp2* mRNA in the PFC (one way ANOVA: F_2,28_ = 14.47, *P* < 0.0001). **G** *Tgfb1* mRNA in the PFC (one way ANOVA: F_2,28_ = 13.49, *P* < 0.0001). **H** *Tgfbr2* mRNA in the PFC (one way ANOVA: F_2,28_ = 12.14, *P* = 0.0002). The data represent mean ± SEM (*n* = 10 or 11). *P < 0.05, **P < 0.01, ***P < 0.001.

To identify the novel targets for the prophylactic effects of (*R*)-ketamine, we performed RNA-sequencing analysis of PFC samples 2 days after CRS (7 days) (Fig. 1A). Using IPA (Ingenuity Pathway Analysis), we analyzed the data of RNA-seq analysis in the PFC. However, we did not detect the pathway(s) which may play a role in the prophylactic effects of (*R*)-ketamine in the PFC although we found a number of differentially expressed genes between the two groups. Therefore, we focused on miRNAs as novel targets for the prophylactic effects of (*R*)-ketamine. We identified 32 sets of miRNAs that expressed differentially in the PFC (Fig. 2A, B). Among miRNAs, we selected miR-132-5p since it has the maximum P-value (Fig. 2B). Using the TargetScanMouse database (www.targetscan.org), we found two genes [brain-derived neurotrophic factor (BDNF) and transforming growth factor β-1 (TGF-β1)] which miRNA-132-5p can regulate (Fig. 2C). It is reported that BDNF and TGF-β1 play a role in the antidepressant-like effects of (*R*)-ketamine in rodents [25, 30, 32, 56, 58]. TGF-β1 binds to TGFBR1 (transforming growth factor beta receptor 1) and TGFBR2 (transforming growth factor beta receptor 2) [32]. Methyl CpG binding protein 2 (MeCP2) is also known to be transcription repressor of BDNF [58–61].

Next, we measured the expression of several genes and miRNA (*Bdnf, Mecp2, Tgfb1, Tgfbr1, miR-132-5p*) in the PFC. We found increased expression of *miR-132-5p* and *Mecp2*, decreased expression of *Bdnf*, *Tgfb1* and *Tgfbr1* in the PFC from CRS-treated mice (Fig. 2D–H). Pretreatment with (*R*)-ketamine (10 mg/kg) significantly attenuated the altered expression of these genes in the PFC of CRS-exposed mice (Fig. 2D–H).

**Fig. 3:**
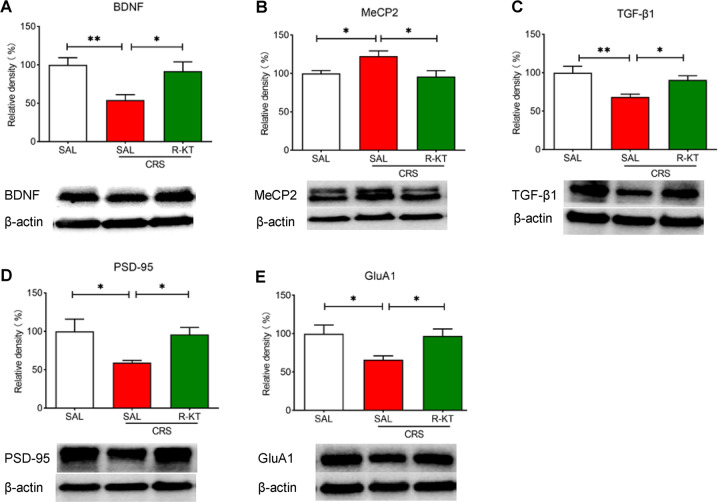
Prophylactic effects of (*R*)-ketamine on the expressions of proteins regulated by miR-132-5p and synaptic proteins in the PFC from CRS-exposed mice. **A** The protein expression of brain-derived neurotrophic factor (BDNF) (one way ANOVA: F_2,27_ = 6.985, *P* = 0.004). **B** Methyl-CpG-binding protein 2 (MeCP2) (one way ANOVA: F_2,27_ = 5.530, *P* = 0.010). **C** Transforming growth factor (TGF)-β1 (one way ANOVA: F_2,27_ = 7.523, *P* = 0.003). **D** Postsynaptic protein-95 (PSD-95) (one way ANOVA: F_2,27_ = 4.888, *P* = 0.015). **E** AMPA Receptor 1 (GluA1) (one way ANOVA: F_2,27_ = 4.825, *P* = 0.016) in the prefrontal cortex (PFC). The data represent mean ± SEM (*n* = 9–11). *P < 0.05, **P < 0.01, ***P < 0.001.

Western blot analysis showed that (*R*)-ketamine (10 mg/kg) significantly improved the reduced levels of BDNF and TGF-β1 in the PFC from CRS-exposed mice, and attenuated the increased expression of MeCP2 (Fig. 3A–C). Furthermore, pretreatment with (*R*)-ketamine significantly ameliorated the reduction of synaptic proteins PSD-95 and GluA1 in the PFC of CRS-exposed mice (Fig. 3D, E)

### Effects of miR-132-5p interferences on CRS-induced depression-like phenotype in mice

**Fig. 4:**
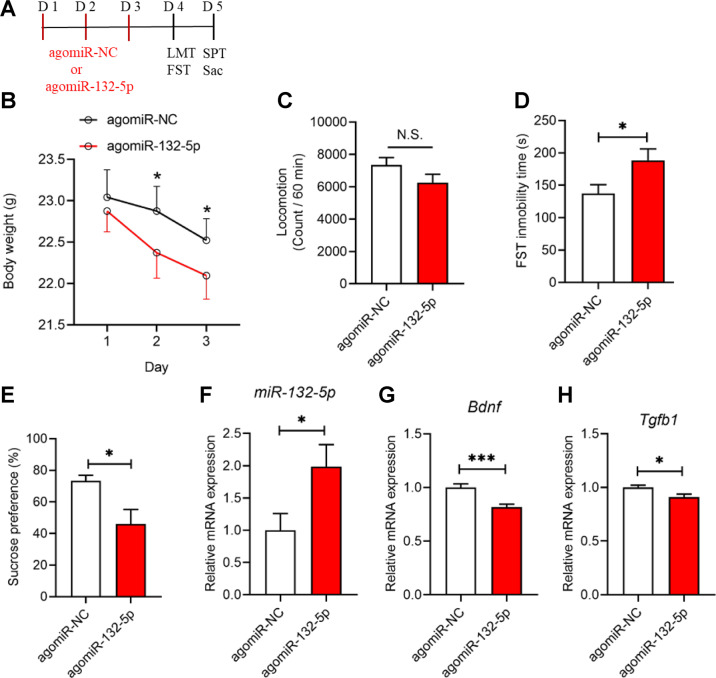
Effects of miR-132-5p agomir on depression-like phenotype. **A** Treatment schedule. AgomiR-132-5p (200 nmol, 2 μl/day) or agomiR-NC (control: 2 μl/day) was administered i.c.v. to mice for 3 days (day 1–day 3). Locomotion test (LMT), forced swimming test (FST) and sucrose preference test (SPT) were performed after the final injection of agomiR-NC or agomiR-132-5p (day 4 and day 5). The prefrontal cortex (PFC) was collected after SPT. **B** Body weight change (two-way repeated measures ANOVA: F_2,36_ = 3.765, *P* = 0.033). **C** Locomotion test (unpaired Student t-test: *P* = 0.129). **D** FST (unpaired Student t-test: *P* = 0.034). **E** SPT (unpaired Student t-test: *P* = 0.012). **F** Expression of *miR-132-5p* in the PFC (unpaired Student t-test: *P* = 0.033). **G** *Bdnf* mRNA in the PFC (unpaired Student t-test: *P* = 0.0005). (**H**): *Tgfb1* mRNA in the PFC (unpaired Student t-test: *P* = 0.018). The data represent mean ± SEM (*n* = 10). *P < 0.05, **P < 0.01, ***P < 0.001. N.S., not significant.

**Fig. 5:**
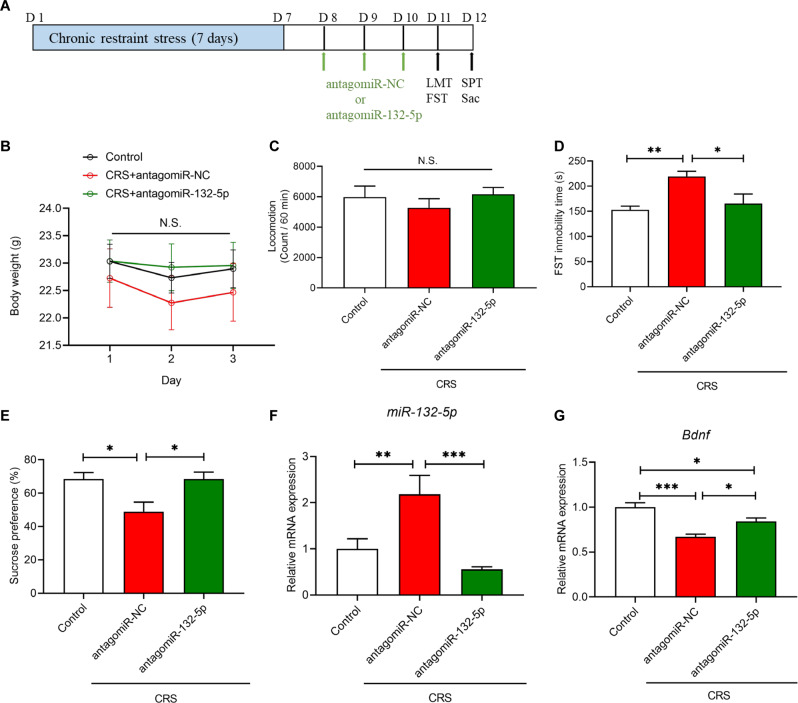
Effects of miR-132-5p knockdown on depression-like phenotype in CRS-induced mice. **A** Treatment schedule. Chronic restraint stress (CRS) was performed 7 days (day 1–day 7). Subsequently, antagomiR-132-5p (200 nmol, 2 μl/day) or antagomiR-NC (control: 2 μl/day) was administered i.c.v. to mice 3 days after CRS (day 8–day 10). Locomotion test, forced swimming test (FST) and sucrose preference test (SPT) were performed after the injection of antagomiR-NC or antagomiR-132-5p (day 11 and day 12). The prefrontal cortex (PFC) was collected after SPT. **B** Body weight change (two-way repeated measures ANOVA: F_4,52_ = 1,125, *P* = 0.300). **C** Locomotion test (one-way ANOVA: F_2,26_ = 0.587, *P* = 0.563). **D** FST (one-way ANOVA: F_2,26_ = 6.709, *P* = 0.005). **E** SPT (one-way ANOVA: F_2,26_ = 5.581, *P* = 0.010). **F** Expression of *miR-132-5p* in the PFC (one-way ANOVA: F_2,26_ = 10.470, *P* = 0.0005). **G** *Bdnf* mRNA in the PFC (one-way ANOVA: F_2,26_ = 15.740, *P* < 0.0001). The data represent mean ± SEM (*n* = 9 or 10). *P < 0.05, **P < 0.01, ***P < 0.001. N.S., not significant.

To further study the role of miR-132-5p in the prophylactic effects of (*R*)-ketamine in CRS-exposed mice, we used agomiR-132-5p and antagomiR-132-5p (Figs. 4A and 5A). RT-PCR was conducted first to verify the efficiency of the miRNA interference. The results showed that the expression of *miR-132-5p* in the PFC was significantly upregulated by agomiR-132-5p and downregulated by antagomiR-132-5p (Figs. 4F and 5F), suggesting that interfering the miRNAs with agonist or antagonist is efficient.

Body weight of mice was significantly decreased after i.c.v. administration of agomiR-132-5p (Fig. 4B). Treatment with agomiR-132-5p increased the immobility time of FST (Fig. 4D) and decreased sucrose preference of SPT (Fig. 4E), without significant effect on locomotion (Fig. 4C). The gene expressions of *Bdnf* and *Tgfb1* in the PFC were significantly decreased in agomiR-132-5p treated mice (Fig. 4G, H). These data show that i.c.v. injections of agomiR-132-5p could decrease the gene expression of *Bdnf* and *Tgfb1* in the PFC of control mice, resulting in depression-like behaviors.

Next, we examined the effects of antagomiR-132-5p on CRS-induced depression-like behaviors (Fig. 5A). The i.c.v. injections of antagomiR-132-5p did not alter body weight and locomotion in CRS-exposed mice (Fig. 5B, C). The i.c.v. injections of antagomiR-132-5p significantly attenuated CRS-induced increased immobility time of FST and decreased sucrose preference of SPT compared to the control (antagomiR-NC) group (Fig. 5D, E). The antagomiR-132-5p significantly blocked the increased expression of *miR-132-5p* in the PFC of CRS-exposed mice (Fig. 5F). Furthermore, the antagomiR-132-5p significantly attenuated the decreased expression of *Bdnf* in the PFC of CRS-exposed mice (Fig. 5G).

These data show that, similar to (*R*)-ketamine, antagomiR-132-5p can elicit prophylactic effects for depression-like behaviors in CRS-exposed mice.

## Discussion

The main findings of this study are as follows: First, pretreatment with (*R*)-ketamine (10 mg/kg) could block depression-like behaviors in CRS-exposed mice. In contrast, (*R*)-norketamine and (2 *R*,6 *R*)-HNK did not show prophylactic effects in the same model. Second, RNA-seq data identified miR-132-5p as the most differentially expressed miRNAs in the PFC for prophylactic effects of (*R*)-ketamine. RT-PCR revealed altered expressions of *miR-132-5p* and its regulated genes (*Bdnf, Mecp2, Tgfb1, and Tgfbr2*) in the PFC of CRS-exposed mice. Furthermore, (*R*)-ketamine significantly ameliorated the altered expression of these genes in the PFC of CRS-exposed mice. Moreover, (*R*)-ketamine significantly ameliorated the altered expression of BDNF, MeCP2, TGF-β1, PSD-95 and GluA1 in the PFC of CRS-exposed mice. Third, i.c.v. injections of agomiR-132-5p caused depression-like behaviors through reduced expression of *Bdnf* and *Tgfb1* mRNA in the PFC of control mice, indicating a role of miR-132-5p in depression-like behaviors. Fourth, i.c.v. injections of antagomiR-132-5p significantly attenuated depression-like behaviors through the improvement of reduced expression of *Bdnf* mRNA in the PFC of CRS-exposed mice. Taken all together, it is likely that miR-132-5p in the PFC plays a role in depression-like behaviors, and that (*R*)-ketamine can exert persistent prophylactic antidepressant-like effects by decreasing miR-132-5p in the PFC.

Here, we found that (*R*)-ketamine, but not its metabolites [(*R*)-norketamine and (2 *R*,6 *R*)-HNK], produced a sustained prophylactic effect in CRS-induced depression-like behaviors in mice. We previously reported that (*R*)-norketamine and (2 *R*,6 *R*)-HNK did not show antidepressant and prophylactic effects in LPS-induced inflammation, LH, and CSDS models of depression [28, 44, 62]. Therefore, it is likely that (*R*)-ketamine itself, but not these metabolites, could have persistent prophylactic effects in CRS-exposed mice.

Wan et al. [54] reported that miR-29b-3p in the PFC plays a key role in the antidepressant-like effects of (*R,S*)-ketamine in chronic unpredictable mild stress model. Subsequently, Huang et al. [55] reported that miR-98-5p in the PFC and hippocampus plays a crucial role in the antidepressant-like effects of (*R,S*)-ketamine in CSDS model. These reports suggest that miR-29b-3p and miR-98-5p might play a role in the antidepressant-like effects of (*R,S*)-ketamine on depression-like behaviors in mice exposed to chronic stress. From our data for prophylactic effects of (*R*)-ketamine, we did not find these miRNAs as differentially expressed miRNAs. Therefore, it is likely that miRNAs for prophylactic effects of (*R*)-ketamine may be different from miRNAs for antidepressant effects of (*R,S*)-ketamine although further study is needed. Considering the crucial role of miRNAs in brain functions, it seems that miR-132-5p in the PFC could play a crucial role in depression-like behaviors as well as sustained prophylactic effects of (*R*)-ketamine. Despite of very short half-life (<30 min) of (*R*)-ketamine in rodents [27], (*R*)-ketamine can elicit long-lasting (>7 days) prophylactic effects in LPS model [44] and CRS model [this study]. It is reported that inhibition of miR-132-5p increased cell survival ability and reduced MPTP (1-methyl-4-pheny-1,2,3,6-tetrahydropyridine)-induced apoptosis of SH-SY5Y cells, suggesting that miR-132-5p inhibition may be a potential therapeutic target for Parkinson’s disease [63]. Collectively, it seems that higher expression of miR-132-5p in the PFC might contribute to depression-like behaviors in CRS-exposed mice, and that inhibition of miR-132-5p could show antidepressant-like effects in CRS-exposed mice. However, the precise mechanisms underlying (*R*)-ketamine-induced long-lasting reduction of miR-132-5p are currently unknown. Further study on relationship between (*R*)-ketamine and long-lasting alterations of miRNAs such as miR-132-5p is needed.

It is known that miRNAs are critically important in gene regulatory networks. Using the TargetScanMouse, we found two genes *Bdnf* and *Tgfb1* as regulatory genes by miR-132-5p. It is well recognized that BDNF plays a key role in the antidepressant-like effects of (*R,S*)-ketamine and its two enantiomers [25, 30, 56, 58, 64]. Furthermore, we identified TGF-β1 as differentially expressed gene for distinguishing antidepressant-like effects of the two enantiomers in CSDS susceptible mice [32]. Here, we found that i.c.v. injection of agomirR-132-5p caused increased expression of *miR-132-5p*, and decreased expression of *Bdnf* and *Tgfb1* in the PFC, resulting in depression-like behaviors in control mice. Furthermore, we found that i.c.v. injection of antagomirR-132-5p attenuated the increased expression of *miR-132-5p*, and the decreased expression of *Bdnf* in the PFC of CRS-exposed mice. Collectively, it is likely that higher expression of miR-132-5p in the PFC plays a role in depression-like phenotypes, and that blockade of miR-132-5p in the PFC could produce antidepressant-like effect through increased expression of BDNF. Therefore, it seems that inhibition of miR-132-5p would be a prophylactic target for stress-related disorders.

MeCP2 is known to be transcription repressor of BDNF [58–61]. A recent study showed that (*R*)-ketamine could activate BDNF transcription through MeCP2 suppression in microglia, and that (*R*)-ketamine showed antidepressant-like effects in CSDS susceptible mice by activating BDNF as well as by inhibiting MeCP2 [58]. In this study, we found that miR-132-5p can regulate the expression of BDNF and MeCP2 in the PFC. Collectively, it is likely that miR-132-5p might play a role in the prophylactic effects of (*R*)-ketamine in CRS model by activating BDNF as well as by inhibiting MeCP2.

Fang et al. [65] reported that plasma levels of miR-132 in medication-free MDD patients were 2.4-fold higher than control subjects, and that there was a positive correlation between *miR-132* levels and the severity of depression, suggesting that blood levels of miR-132-5p may be a state biomarker for depression. A recent systematic review showed a list of dysregulated circulatory miRNAs in MDD patients as the most prominent miRNAs such as miR-24-3p, miR-26a-5p, miR-135a, miR-425-3p, miR-132, miR-124, and miR-16-5p [50]. In addition, miR-132 is the most frequently upregulated miRNA in MDD patients [49]. Thus, it is likely that miR-132-5p may play a role in depressive symptoms in patients with MDD. It is, therefore, of interest to investigate whether miR-132-5p may be a peripheral biomarker for prophylactic or antidepressant effects of (*R*)-ketamine in patients with psychiatric disorders such as MDD. A recent study identified the red blood cell-specific miR-144-3p as a blood biomarker to aid depression diagnosis and predict treatment response to ketamine [66]. Therefore, it is also interesting to investigate the role of miR-144-3p in action of ketamine and its enantiomers.

Patients with depression have high rate of relapse/recurrence. The relapse of depressive symptoms after successful treatment in MDD patients is approximately 50% or more within six months [67]. Considering potent sustained prophylactic effects of (*R*)-ketamine, it is likely that (*R*)-ketamine may prevent the relapse in depressed patients. It is, therefore, of interest to investigate whether (*R*)-ketamine can reduce the recurrent rate in patients with MDD, BD, or PTSD.

This study has some limitations. First, we did not examine whether agomiR-132-5p can block prophylactic effects of (*R*)-ketamine in CRS model. Furthermore, we did not examine the effects of (*R*)-norketamine and (2 *R*,6 *R*)-HNK on increased expression of miR-132-5p in the PFC of CRS model. Further study of these experiments is needed to confirm the role of miR-132-5p in the prophylactic actions of (*R*)-ketamine. Second, AMPAR (α-amino-3-hydroxy-5-methyl-4-isoxazolepropionic acid receptor) plays a role in the antidepressant-like actions of (*R*)-ketamine in CSDS susceptible mice [25]. However, we did not examine the role of AMPAR in the regulation of miR-132-5p by (*R*)-ketamine. Further study is needed to examine the effects of AMPAR antagonist on gene expression of miR-132-5p by (*R*)-ketamine. Finally, we did not examine the role of miR-6928-5p in the prophylactic effects of (*R*)-ketamine although further study is needed.

In conclusion, this study shows that increased expression of miR-132-5p in the PFC might contribute to depression-like behaviors in CRS-exposed mice, and that inhibition of miR-132-5p might play a role in the persistent prophylactic effects of (*R*)-ketamine. Therefore, it is of interest to investigate whether miRNAs such as miR-132-5p play a role in antidepressant or prophylactic effects of (*R*)-ketamine in depressed patients with MDD, BD, or PTSD.

## Data Availability

The RNA sequencing data have been deposited to the NCBI Sequence Read Archive and are available at the accession number PRJNA843925.
